# Phase I trial of docetaxel, cisplatin and concurrent radical radiotherapy in locally advanced oesophageal cancer

**DOI:** 10.1038/sj.bjc.6606051

**Published:** 2010-12-14

**Authors:** F L Day, T Leong, S Ngan, R Thomas, M Jefford, J R Zalcberg, D Rischin, J McKendick, A D Milner, J Di Iulio, A Matera, M Michael

**Affiliations:** 1 Department of Medical Oncology, Peter MacCallum Cancer Centre, Locked Bag 1, A’Beckett St, Melbourne, Victoria 8006, Australia; 2 Department of Radiation Oncology, Peter MacCallum Cancer Centre, Locked Bag 1, A’Beckett St, Melbourne, Victoria 8006, Australia; 3 Division of Surgical Oncology, Peter MacCallum Cancer Centre, Locked Bag 1, A’Beckett St, Melbourne, Victoria 8006, Australia; 4 Department of Medical Oncology, Box Hill Hospital, Box Hill, Australia; 5 Centre for Biostatistics and Clinical Trials, Peter MacCallum Cancer Centre, Locked Bag 1, A’Beckett St, Melbourne, Victoria 8006, Australia

**Keywords:** oesophageal cancer, chemoradiotherapy, docetaxel, cisplatin

## Abstract

**Background:**

Locally advanced oesophageal cancer (LAEC) is associated with poor survival and more effective treatments are needed. The aim of this phase I trial was to assess the maximum tolerated dose (MTD) of a novel weekly docetaxel and cisplatin regimen concurrent with radical radiotherapy.

**Methods:**

Patients with unresectable, non-metastatic LAEC were eligible. Treatment comprised docetaxel 15–30 mg m^−2^ per week and cisplatin 15–30 mg m^−2^ per week in six planned dose levels (DLs) in 3–6 patient cohorts with 50 Gy radiotherapy in 25 fractions. Maximum tolerated dose was based on defined dose-limiting toxicities (DLTs) during therapy and 2 weeks post therapy.

**Results:**

A total of 24 patients were enrolled. There were two DLTs: grade 3 fever in DL1 (docetaxel 15 mg m^−2^, cisplatin 15 mg m^−2^) and grade 3 nausea in DL2 (20 mg m^−2^, 15 mg m^−2^). These DLs were each expanded to six patients without further DLTs. The most common acute toxicity was grade 3 radiation oesophagitis (37.5%). There were no grade 4 toxicities, and haematologic toxicity was minimal. Cisplatin and docetaxel dose intensity was 100% at the highest dose level (DL6). A MTD was not reached in this trial. Tumour overall response rate was 50% (33% complete, 17% partial).

**Conclusion:**

Cisplatin and docetaxel each 30 mg m^−2^ per week concurrent with 50 Gy radiotherapy is recommended for use in phase II clinical trials in oesophageal cancer.

Oesophageal cancer is an important global health problem, ranking 7th among male cancer deaths in the United States and increasing in incidence (Jemal *et al*, 2009). The 5-year survival rates are poor, approximately 17% in the period 1996–2004 (Jemal *et al*, 2009). Surgical resection, with neoadjuvant chemotherapy or chemoradiotherapy (Gebski *et al*, 2007), confers the greatest likelihood of long-term survival, but only 30–40% of patients with newly diagnosed oesophageal cancer have resectable disease. For patients with unresectable, locally advanced oesophageal cancer (LAEC) (due to advanced T or N stage, or patient medically unsuitable for surgery), chemoradiotherapy is the mainstay of treatment.

The most commonly used chemotherapy for radiosensitisation in LAEC, and in palliation, are the platinums and 5-fluorouracil. The RTOG 85–01 trial randomised patients with LAEC to receive cisplatin and infusional 5-fluorouracil concurrently with 50 Gy or 64 Gy radiotherapy alone. This study showed a significantly improved 5-year survival of 27% (*vs* 0%) for patients receiving chemoradiation (Herskovic *et al*, 1992; Al-Sarraf *et al*, 1997). Nevertheless, outcomes were still poor in the combined modality arm, with 25% patients having persistent disease, and 2-year rates of local recurrence and distant disease of 45 and 21%, respectively. New active therapies and treatment approaches in oesophageal cancer are warranted.

Docetaxel is a semi-synthetic taxane with promising single-agent activity in advanced oesophageal cancer (Einzig *et al*, 1996), and greater activity in *in vitro* oesophageal cancer studies than paclitaxel (Kawamura *et al*, 1997). Docetaxel is a potent radiosensitiser through promotion of microtubule stability, causing arrest in G2 and M phases of the cell cycle and, hence, sensitivity to radiation injury (Mason *et al*, 1997). A phase I trial of docetaxel concurrent with 60 Gy thoracic radiotherapy in patients with oesophageal and non-small-cell lung cancer (NSCLC) (Mauer *et al*, 1998) found the maximum tolerated dose (MTD) of docetaxel to be 20 mg m^−2^ per week. The dose limiting toxicity (DLT) for the weekly regimen in this study was grades 3 and 4 oesophagitis. Local and systemic disease control could potentially be enhanced by the addition of cisplatin, given its known activity in oesophageal cancer and non-overlapping toxicity profile.

In this phase I trial, we assessed a novel regimen of weekly docetaxel and cisplatin concurrent with radiotherapy (50 Gy) for patients with LAEC. The primary objective was to define the MTD of this chemotherapy combination with concurrent radical radiotherapy, with a view to establishing a recommended dose level for future multimodality clinical trials. The secondary objectives were to define the safety (acute and long term) of this regimen when combined with radical radiotherapy, as well as determine the response rate, overall survival (OS) and progression-free survival (PFS) of the patients treated with this protocol.

## Patients and methods

### Patients

Patients were eligible if they met the following criteria: (1) histologically proven squamous cell carcinoma or adenocarcinoma of the oesophagus; (2) considered unsuitable for surgical resection (due to advanced T or N stage, or patient medically unsuitable for surgery) based on multidisciplinary opinion; (3) no previous therapy for oesophageal cancer; (4) ECOG performance status (PS) of zero or one; (5) life expectancy >3 months; (6) adequate organ function – (i) hepatic: serum bilirubin ⩽1.0 × upper limit normal (ULN), AST and/or ALT ⩽2.0 × ULN, ALP ⩽2.5 × ULN, (ii) bone marrow: haemoglobin ⩾100g l^−1^, neutrophil count ⩾1.5 × 10^9^ per l, platelet count ⩾100 × 10^9^ per l, (iii) renal: creatinine clearance ⩾55 ml min^−1^ (using radioisotope renal scan or derived from serum creatinine using the Cockroft-Gault formula); (7) agreed compliance to adequate contraception; (8) written informed consent.

The following patients were ineligible: (1) resectable oesophageal carcinoma; (2) carcinoma of the cervical oesophagus; (3) tumour predominantly in the stomach; (4) metastatic oesophageal carcinoma; (5) medical comorbidities which would compromise the delivery of therapy or which may be exacerbated by the planned treatment; (6) receiving treatment with another investigational agent; (7) pregnant or lactating females.

Institutional ethics committee approval was obtained from each participating site.

### Treatment plan

#### Radiation therapy

External beam radiotherapy was given to a total dose of 50 Gy in 25 fractions, five fractions per week for 5 weeks. Treatment was delivered using conformal techniques in accordance with ICRU 50/62 recommendations. In most cases, a two-phase technique was employed, comprising AP-PA fields to a dose of 36 Gy in 18 fractions, followed by lateral fields to a dose of 14 Gy in 7 fractions. The gross tumour volume (GTV) comprised the primary tumour and involved lymph nodes as defined by imaging, endoscopy and biopsy. The clinical target volume (CTV) was generated by applying standard margins (5 mm radially and 4 cm longitudinally) to the GTV, and the planning target volume (PTV) was then generated by applying a volumetric 10 mm margin.

#### Concurrent chemotherapy

During radiotherapy, all patients received intravenous docetaxel and cisplatin administered weekly (days 1, 8, 15, 22 and 29). Chemotherapy doses were escalated through six planned dose levels (DLs) as shown in Table 1. Chemotherapy was given within 4 h before the delivery of radiation that day. Steroid and anti-emetic pre-medication was administered to all patients.

#### Definition of dose-limiting toxicities (DLTs)

The following toxicities (as per NCI-CTC version 2.0) occurring during or up to 2 weeks after chemoradiotherapy were defined prospectively as DLTs: (1) grade 4 neutropaenia (neutrophil count <0.5 × 10^9^ per l) of any duration; (2) grade 3 neutropaenia of any duration during chemoradiotherapy; (3) grade 4 thrombocytopaenia (platelet count <10 × 10^9^ per l) of any duration; (4) grade 3 thrombocytopaenia (10–49 × 10^9^ per l) with bleeding; (5) febrile neutropaenia; (6) grade 4 radiation oesophagitis; (7) any non-oesophagitis grade 3 or 4 radiotherapy toxicity; (8) any clinically significant treatment-related grade 3 or 4 toxicity outside the radiotherapy field, with the exception of alopecia; (9) interruption of radiotherapy for >1 week; (10) omission of chemotherapy for ⩾1 week; (11) toxicity requiring ⩾2 chemotherapy dose reductions.

#### Dose escalation schema

Three patients were entered into each DL. If no DLTs were observed, the next DL was opened. If a DLT was observed in one of three patients, then three additional patients (a total of six) were accrued at this level. If DLTs were observed in one of six patients, then escalation to the next level took place. If DLTs were observed in ⩾2 of 3 or ⩾2 of 6 patients, then no further dose escalation took place. Maximum tolerated dose was predefined as the second highest dose level reached.

Dose escalation to the next DL, or expansion of the current DL, only occurred when all three patients at the current DL had completed chemoradiotherapy and 2 weeks post-therapy. There was no intra-patient dose escalation.

#### Dose modifications during chemoradiotherapy

Radiotherapy, together with chemotherapy, was suspended if the patient experienced grade 4 oesophagitis, or grade 3 or 4 other radiation-associated toxicity. Treatment could recommence once reactions had improved to grade 1, unless treatment was interrupted for 2 or more weeks, in which case all therapy was to be ceased.

Prospectively defined chemotherapy dose modifications (dose omission, delay or reduction) were based on the worst grades of haematological and non-haematological toxicity during chemoradiotherapy. All dose reductions were permanent. Treatment was ceased if grade 3 or 4 neurological toxicity, other grade 4 non-haematological toxicity, more than one dose reduction required in DL1, or more than two dose reductions required in higher DLs. Radiotherapy continued despite chemotherapy modifications.

### Monitoring procedures and tests

At baseline (within 1 week of study entry), patients underwent physical examination, assessment of PS, and blood taken for full blood examination (FBE), biochemistry (including serum urea, creatinine, electrolytes, calcium, liver function tests (bilirubin, AST, ALT, ALP, GGT)) and determination of creatinine clearance. Within 4 weeks before study entry, patients underwent endoscopy, computed tomography (CT) scan of the chest/abdomen and whole-body bone scan. Optional baseline tests were endoscopic ultrasound, bronchoscopy and FDG-PET scan (subject to availability).

During treatment, patients were reviewed weekly, had their weight measured, PS assessed, physical examination and recording of acute toxicities. Blood was taken for FBE twice per week, and biochemistry and creatinine clearance measured weekly.

Following completion of chemoradiotherapy, patients were assessed clinically (physical examination, body weight and PS) at weeks 1, 2, 4 and 6. Full blood examination and biochemistry were measured at weeks 1 and 2. Patients underwent repeat endoscopy and CT scan of the chest/abdomen at 6 weeks, and FDG-PET at 8–10 weeks (subject to availability).

Follow-up evaluation then occurred every 3 months until patient death or loss to follow-up. This comprised clinical examination, assessment of late radiation toxicity and repeat CT scan of chest/abdomen.

### Statistical methods

Acute toxicity was graded and reported according to the NCI-CTC version 2.0 (30 April 1999). Late radiation toxicities were assessed using the RTOG/EORTC criteria. RECIST version 1.0 was used for assessment of radiological response.

Progression-free survival was measured from treatment start date until disease progression or death from any cause. Overall survival was measured from treatment start date until death from any cause. Progression-free survival and OS were determined using the Kaplan–Meier method, with times censored at the close-out date for patients still being followed without evidence of disease/death, or the date of last contact for those patients lost to follow-up. Using Logit transformation 95% confidence intervals were calculated. All other data were summarised using descriptive statistics, including counts and percentages for categorical data and the median and range for continuous data. Analyses were conducted using StatXact (Version 5.0.3, Cytel Software Corporation, Cambridge, MA, USA; 2001) and S-Plus 2000 (MathSoft Inc., Seattle, WA, USA; 1999).

## Results

### Patients

A total of 24 patients were recruited from three centres in Melbourne, Australia, between May 2001 and January 2007; Peter MacCallum Cancer Centre, Box Hill Hospital and Monash Medical Centre. The majority of patients were ineligible for surgical resection on the basis of nodal involvement. Of the optional staging tests, 11 patients received a baseline FDG-PET scan, 4 patients underwent endoscopic ultrasound and 3 patients bronchoscopy. Patient demographics are summarised in Table 2. Median patient age was 58 years and males predominated (92%). One patient had undergone previous surgery for oesophageal carcinoma.

### Treatment delivery

#### Radiotherapy

A total of 23 patients (96%) completed all planned radiotherapy without any treatment interruptions. One patient did not complete the final two fractions of radiotherapy because of grade 3 radiation oesophagitis and fever. Three patients received 50.4 Gy in 28 fractions, rather than the protocol specified 50 Gy in 25 fractions, because of investigator prescription error.

#### Chemotherapy

The 24 accrued patients were treated across six DLs as shown in Table 1. DLs one and two were each expanded to six patients because of the occurrence of a DLT in one of the three initial patients.

Only one patient did not receive the protocol-defined chemotherapy doses. This patient was in DL3 and had cisplatin omitted on days 15 and 22 because of creatinine clearance <55 ml min^−1^ on those dates. No docetaxel doses were omitted. One patient (DL1) had a 2-day delay in chemotherapy administration because of hospitalisation with a DLT. The relative dose intensity (ratio of actual dose to planned dose) in the highest dose level (DL6) was 100% for both cisplatin and docetaxel.

#### Dose-limiting toxicities

Two DLTs were observed. One patient in DL1 experienced grade 3 fever, without neutropaenia. This patient was concurrently suffering from grade 3 oesophagitis, and was hospitalised for intravenous antibiotics and analgesia. This event was the cause of the only delay in chemotherapy administration and the only omission of radiotherapy. Dose level 1 was expanded to six patients as a result, but no further DLTs were observed.

The second DLT was in DL2, and consisted of grade 3 nausea despite anti-emetic treatment. Modification of chemoradiotherapy was not indicated, but DL2 also expanded to six patients. No further DLTs were observed, and hence a MTD was not defined.

### Toxicity

There were two grade 3 haematologic toxicity events; both grade 3 leucopaenia. The first event was in DL2 and occurred at day 40 (after completion of chemoradiotherapy). The second was in DL6 and occurred at day 30, one day after final chemotherapy dosing.

Non-haematologic toxicity is shown in Table 3. In all, 37.5% of patients experienced grade 3 dysphagia attributed to radiation oesophagitis. There were no episodes of grade 4 oesophagitis and no acute pulmonary toxicity. Outside the radiotherapy field the most common adverse event was fatigue.

Late radiation toxicities are shown in Table 4.

### Response

In all, 21 of 24 patients were evaluable for radiologic response within the radiation field (Table 5). The within-field response rate was 54% (37% complete and 17% partial), and 21% of patients had stable disease. Three patients (13%) had progressive disease within the radiotherapy field, all concurrent with distant progression. The overall response rate to treatment, incorporating systemic disease progression, was 50% (Table 5).

A total of 11 patients underwent FDG-PET at baseline, and 10 of these also progress scans following treatment. Four patients experienced a complete metabolic response (40%), five a partial metabolic response (50%) and one patient progressive disease (10%).

### Further treatment post-chemoradiotherapy

Six patients (25%) later underwent oesophageal surgery with curative intent, range 56–283 days after completing chemoradiotherapy. Surgery performed was Ivor–Lewis oesophagectomy in four patients, three-stage oesophagectomy in one patient and total oesophagectomy in one patient.

### Sites of relapse

**Figure 1:**
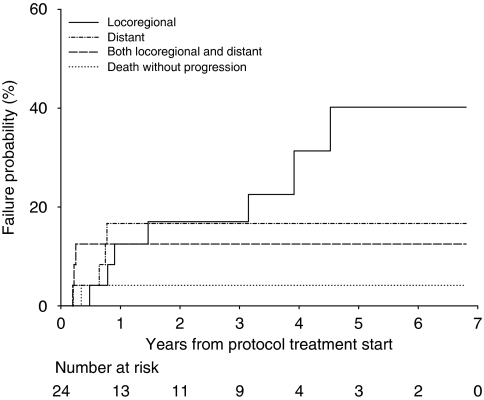
Site of first failure in patients with recurrent disease (*n*=14).

Patients were followed to a close-out date of 16 June 2010. One patient was lost to follow-up after completing protocol treatment and hence was not evaluable for treatment response, but has been included in survival analyses as the date of death is known. Median patient follow-up was 5.2 years. Disease relapse was reported in 14 patients. The location of first disease progression is shown in Figure 1. In all, 42% of enrolled patients (10 of 24) experienced initial local disease relapse (primary site and/or regional nodes) and 29% (7 of 24) distant metastases.

### Survival parameters

**Figure 2:**
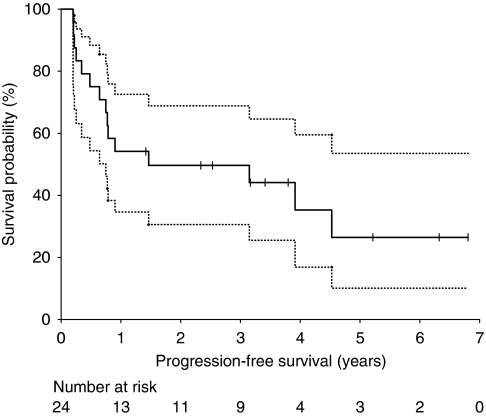
Kaplan–Meier curves for progression-free survival for all patients. Dotted lines represent the 95% CI. Vertical lines represent patients censored at the close-out date.

**Figure 3:**
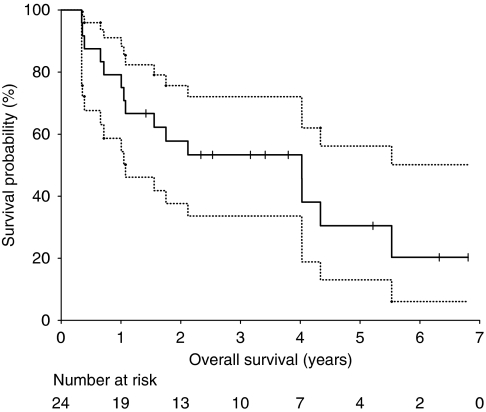
Kaplan–Meier curves for overall survival for all patients. Dotted lines represent the 95% CI. Vertical lines represent patients censored at the close-out date.

Kaplan–Meier estimates of PFS are shown in Figure 2. Median PFS was 1.46 years (95% CI 0.75–6.81 years). Progression-free survival at 2 years was 49.7% (95% CI 30.6–68.8%), and 26.5% at 5 years (95% CI 10.1–53.5%). Median OS was 4.02 years (95% CI 1.08–6.81 years, Figure 3). Overall survival at 2 years and 5 years was 57.8% (95% CI 37.6–75.6%) and 30.5% (95% CI 13.1–56.2%), respectively.

## Discussion

The semi-synthetic taxane docetaxel has shown activity in oesophageal cancer in numerous clinical trials. Docetaxel has been used in advanced oesophageal cancer as a single agent (Einzig *et al*, 1996; Heath *et al*, 2002), and in combination with cisplatin (Laack *et al*, 2005), 5-fluorouracil (Chun *et al*, 2001), capecitabine (Lorenzen *et al*, 2005) and irinotecan (Lordick *et al*, 2003). Known to be a potent radiosensitiser, docetaxel has also been used in chemoradiotherapy for oesophageal cancer (Mauer *et al*, 2000; Pasini *et al*, 2005; Font *et al*, 2007; Ruhstaller *et al*, 2009). The initial phase I trial of single agent docetaxel with thoracic radiotherapy (Mauer *et al*, 1998) included nine patients with oesophageal cancer (total *n*=29, remainder NSCLC) and tested docetaxel in escalating total doses of 40, 60 or 75 mg m^−2^ (given in one, two or three divided doses) per 21-day cycle and concurrent with median 60 Gy radiotherapy. Dose-limiting toxicities in this trial were radiation oesophagitis and febrile neutropaenia; the docetaxel MTD was 20 mg m^−2^ per week. This docetaxel dose and schedule was then tested in a phase II trial (Font *et al*, 2007) in 34 patients with oesophageal cancer unsuitable for surgical resection based on T4 status or co-morbidities, and given concurrently with 66 Gy radiotherapy. Radiologic response rates were 26% complete and 24% partial. Median OS was 6 months, with 35% and 12% patients alive at 1 and 3 years, respectively. The rate of grade 3 and 4 radiation oesophagitis was 17%. These phase I and II trials (Mauer *et al*, 1998; Font *et al*, 2007) of single agent docetaxel with 60–66 Gy radiotherapy each had two deaths from radiation pneumonitis.

Chemoradiotherapy using both docetaxel and cisplatin has been the subject of phase I trials in NSCLC. Although the DLT for each published study was radiation oesophagitis, variable MTDs are reported; docetaxel 20 mg m^−2^ per week with cisplatin fixed at 20 mg m^−2^ per week and 63 Gy radiotherapy (Wu *et al*, 2002), docetaxel 25 mg m^−2^ per week with cisplatin fixed at 25 mg m^−2^ per week and 60 Gy (Mudad *et al*, 2003), and docetaxel and cisplatin both at 40 mg m^−2^ per week with 60 Gy (Kiura *et al*, 2003). The latter study was expanded to enroll 42 patients in the phase II setting, and found an excellent response rate of 79% in stage III NSCLC, but high rates of grade 3 and 4 myelosuppression (Kiura *et al*, 2003). A subsequent study by the same authors used this regimen (cisplatin 40 mg m^−2^ per week, docetaxel 40 mg m^−2^ per week, 60 Gy radiotherapy) as induction treatment before resection in locally advanced NSCLC, and found that 11 of 22 patients (50%) were unable to complete chemotherapy without dose modification because of toxicity (Katayama *et al*, 2004).

Pasini *et al* (2005) conducted a phase I study in localised oesophageal cancer using induction weekly docetaxel, cisplatin and infusional 5-fluorouracil, followed by escalating doses of the same three agents concurrent with up to 50 Gy radiotherapy, then surgery. Maximum tolerated dose was not reached at the highest planned dose level; docetaxel 35 mg m^−2^ per week, cisplatin 25 mg m^−2^ per week and infusional 5-FU 150 mg m^−2^ per day with 50 Gy radiotherapy. These doses are the subject of an ongoing phase II trial by the same group. A recently published Swiss multicentre phase II trial used induction docetaxel and cisplatin (75 mg m^−2^ each, two 21-day cycles), followed by weekly concurrent treatment (20 mg m^−2^ and 25 mg m^−2^, respectively) with 45 Gy radiotherapy, then surgery, in locally advanced but resectable oesophageal cancer (Ruhstaller *et al*, 2009). Concurrent chemoradiotherapy resulted in grade 3 and 4 dysphagia in only 8% of patients and grade 3 and 4 anaemia, thrombocytopenia and neutropenia each in less than 3%. In all, 86% of patients proceeded to surgical resection and R0 resection was achieved in 79% enrolled patients.

The phase I trial reported here used 50 Gy radiotherapy with the goal of optimising systemic therapy delivery and radiosensitisation without dose-limiting radiotherapy toxicity. Cisplatin was added to docetaxel because of its established role as a radiosensitiser in oesophageal cancer and minimal additional myelotoxicity. In all, 9 of 24 patients (37.5%) experienced grade 3 radiation oesophagitis, but there were no episodes of grade 4 oesophagitis. Other than the two reported DLTs, toxicities were low grade, not probably/definitely related to treatment, or deemed not clinically significant. There were no radiation pneumonitis events, and haematologic toxicity from the weekly chemotherapy was negligible, consistent with the known lower myelotoxicity of this docetaxel schedule (Hainsworth *et al*, 1998). The maximum severity late toxicity was grade 2 and skin related. Only one patient did not receive 100% of prescribed chemotherapy and radiotherapy doses because of toxicity, and the maximum protocol cisplatin and docetaxel doses (DL6) were given without achieving a MTD as predefined in the trial protocol, as also observed by Pasini *et al* (2005) with a similar backbone and doses, but with the addition of infusional 5-FU. Like Pasini *et al* (2005), we elected not to increase chemotherapy doses above the pre-planned levels, despite not achieving a MTD, because of expected prohibitive toxicity with further escalation. The highest DL in this trial exceeds the MTDs established in concurrent chemoradiotherapy for NSCLC (Wu *et al*, 2002; Mudad *et al*, 2003), other than the study by Kiura *et al* (2003), which has subsequently shown significant toxicity, and the cisplatin and docetaxel doses are also greater than those used in the phase II oesophageal cancer trial by Ruhstaller *et al* (2009).

Although not powered to demonstrate improvements in cancer outcomes, the radiologic response rate of 54% in the radiotherapy field, and 50% overall, in this trial compares favourably with phase II chemoradiotherapy trials (Font *et al*, 2007; 50%), including those also using induction chemotherapy before docetaxel-based chemoradiotherapy (Mauer *et al*, 2000; 58%). The median PFS and OS of 1.5 and 4.0 years presented here are surprisingly separate, given the median survival time of 8 months with recurrent oesophageal cancer (Meguid *et al*, 2009); this apparent discrepancy is because of the effect of small patient numbers on survival curves. Nonetheless, these survival times are both considerably longer than those of the landmark RTOG 85-01 trial cisplatin/5-fluorouracil/radiotherapy arm (1 year and 14.1 months, respectively). Participants in this study were median age 6 years younger than those in RTOG 85-01, better PS and different in tumour histology (54% *vs* 12% adenocarcinoma) and site (greater proportion lower oesophagus), but significantly higher T and N stage. The subsequent use of curative-intent surgery in six patients (25%) in the current trial may have contributed to the improved survival outcomes. The predominance of locoregional recurrence over distant disease progression in this trial is similar to that seen in the randomized chemoradiotherapy arm of RTOG 85-01, postulated by those authors as reflecting an effect of chemotherapy on micrometastatic disease (Al-Sarraf *et al*, 1997).

In conclusion, the phase I trial presented here has shown that weekly administration of cisplatin and docetaxel to each 30 mg m^−2^ per week concurrent with 50 Gy radiotherapy is tolerable and deliverable in locally advanced oesophageal cancer. These results have led to the incorporation of this chemoradiotherapy backbone into a current phase II study of the Australasian Gastro-Intestinal Trials Group (AGITG) in resectable oesophageal cancer with a randomisation to the addition of cetuximab.

## Figures and Tables

**Table 1:** Chemotherapy dose levels

**Dose level (DL)**	**Docetaxel weekly (mg m^−2^)**	**Cisplatin weekly (mg m^−2^)**	**Participants**
0^a^	10	10	
1	15	15	6
2	20	15	6
3	20	20	3
4	25	20	3
5	30	20	3
6	30	30	3

a Included in protocol for patients who require dose reduction below DL1.

**Table 2:** Patient characteristics

**Characteristics**	**Number (*n*=24)**	**%**
*Sex*		
Male: female	22 : 2	92 : 8
		
*Age (years)*		
Median	58	
Range	36–83	
		
*ECOG performance status*		
0	7	29.2
1	17	70.8
		
*Histology*		
Squamous	11	45.8
Adenocarcinoma	13	54.2
		
*Tumour location*		
Upper third	3	12.5
Middle third	5	20.8
Lower third	16	66.7
		
*T stage*		
1	3	12.5
2	3	12.5
3	16	66.7
4	2	8.3
		
*N stage*		
0	6	25.0
1	18	75.0
		
*Stage grouping*		
I	2	8.3
IIa	4	16.7
IIb	2	8.3
III	16	66.7
		
*Previous surgery*		
Yes	1	4.2
No	23	95.8
		
*Weight loss over previous 3 months*		
None	9	37.5
⩽10%	11	45.8
>10%	4	16.7

Abbreviation: ECOG=eastern cooperative oncology group.

**Table 3:** Non-haematologic toxicities observed during chemoradiotherapy and within 2 weeks after completion, and considered possibly, probably or definitely related to treatment (NCI-CTC Version 2, 30 April 1999)

		**Dose level**						
**Toxicity**	**Worst grade**	**1 (*n*=6)**	**2 (*n*=6)**	**3 (*n*=3)**	**4 (*n*=3)**	**5 (*n*=3)**	**6 (*n*=3)**	**Total (%)**
*Within radiotherapy field*								
Dysphagia	3	2	3	1	0	2	1	9 (37.5)
								
*Outside radiotherapy field*								
Anorexia	3	0	1	0	0	0	0	1 (4.2)
Nausea	3	0	2	0	0	0	1	3 (12.5)
Constipation	3	1	0	1	0	0	1	3 (12.5)
Fatigue	3	1	1	2	0	0	0	4 (16.7)
Fever	3	1^a^	0	0	0	0	0	1 (4.2)
								
*Biochemical*								
Hyperglycaemia	3	0	1	0	0	0	0	1 (4.2)
Hyponatraemia	3	0	1	0	0	1	0	2 (8.3)
Elevated GGT	4	2^b^	0	0	0	1	0	3 (12.5)

a Without neutropenia.

b One patient in DL1 experienced grade 4 elevation of GGT, attributed to penicillin antibiotics, and reversed on their cessation.

**Table 4:** Late radiotherapy toxicities (EORTC/RTOG criteria)

		**Dose level**						
**Toxicity**	**Worst grade**	**1 (*n*=6)**	**2 (*n*=6)**	**3 (*n*=3)**	**4 (*n*=3)**	**5 (*n*=3)**	**6 (*n*=3)**	**Total (%)**
Esophageal	1	4	2	1	0	1	1	9 (37.5)
Skin	2	2	0	0	0	1	0	3 (12.5)
Pulmonary	1	2	2	0	0	0	0	4 (16.7)

**Table 5:** Radiologic response at completion of chemoradiotherapy (RECIST version 1.0)

	**Dose levels**						
**Response parameters**	**1 (*n*=6)**	**2 (*n*=6)**	**3 (*n*=3)**	**4 (*n*=3)**	**5 (*n*=3)**	**6 (*n*=3)**	**No. (%) (Total *n*=24)**
*Overall best response*							
Complete response	4	1	0	2	1	0	8 (33)
Partial response	1	3	0	0	0	0	4 (17)
Stable disease	0	0	3	0	1	1	5 (21)
Progressive disease	1	2	0	0	1	1	5 (21)
Not evaluable^a^	0	0	0	1	0	1	2 (8)
							
*Best response in radiotherapy field*							
Complete response	4	2	0	2	1	0	9 (37)
Partial response	1	3	0	0	0	0	4 (17)
Stable disease	0	0	3	0	1	1	5 (21)
Progressive disease	1	1	0	0	0	1	3 (13)
Not evaluable^b^	0	0	0	1	1	1	3 (13)

a One patient not evaluable by radiologic criteria (DL6), one patient lost to follow-up (DL4).

b Patient in DL5 not evaluable at primary site, progressive disease systemically.
